# Potentially Toxic Metal Levels in Cheeses Produced in Türkiye: Regional Variation, Milk Source, Ripening Stage, Production Type, and Associated Dietary Exposure and Health Risk

**DOI:** 10.1007/s12011-026-05198-z

**Published:** 2026-06-27

**Authors:** Hüseyin Ender Gürmeriç, Mustafa Şengül, Burhan Basaran

**Affiliations:** 1 Department of Food Processing, Şiran Mustafa Beyaz Vocational School, Gümüşhane University, Şiran, Gümüşhane, 29700 Türkiye; 2 Department of Food Engineering, Faculty of Agriculture, Atatürk University, Erzurum, 25240 Türkiye; 3 Department of Nutrition and Dietetics, Faculty of Health Sciences, Recep Tayyip Erdogan University, Rize, 53100 Türkiye

**Keywords:** Food safety, ICP-MS, Hazard index, Toxicological contribution, Non-carcinogenic risk

## Abstract

This study investigated potentially toxic metal (PTM) levels in cheeses produced in different regions of Türkiye and evaluated the associated dietary exposure and non-carcinogenic health risk. A total of 84 cheese samples were collected from seven geographical regions and classified according to milk source, ripening stage, and production type. After microwave-assisted acid digestion, PTM levels were quantified by inductively coupled plasma mass spectrometry (ICP-MS), and exposure and non-carcinogenic risk were evaluated using Estimated Daily Intake (EDI), Target Hazard Quotient (THQ), and Hazard Index (HI). Aluminium (Al), iron (Fe), and zinc (Zn) ranged from <0.00215 to 7.29,<0.00769 to 38.8, and 0.68 to 56.6 mg/kg wet weight, respectively, whereas chromium (Cr), nickel (Ni), copper (Cu), selenium (Se), cadmium (Cd), and lead (Pb) ranged from <0.58 to 244, <1.26 to 369, <1.88 to 4067, <0.91 to 1378, <0.06 to 12.3, and <2.19 to 1754 µg/kg wet weight, respectively. Values marked with “<” represent concentrations below the limit of quantification (LOQ). Arsenic (As) was not detected in any sample. Significant differences were observed for selected PTMs according to ripening stage, milk source, production type, and region (p<0.05). The toxicological contribution (TC) values for nutritional PTM exposure were low. THQ and HI values remained below 1, suggesting a low overall non-carcinogenic risk; however, Pb exceedances in some samples indicate the need for continued PTM monitoring.

**Supplementary Information:**

The online version contains supplementary material available at https://doi.org/10.1007/s12011-026-05198-z.

## Background

Cheese is a nutrient-dense dairy product that provides high-biological-value proteins, calcium, vitamins, essential minerals, and lipids [1]. Its consumption has been associated with reduced risk of cardiovascular disease, coronary heart disease, and stroke, as well as with potential benefits for bone and cognitive health [2]. The natural enzyme and microorganism content of cheese provides a suitable environment for probiotic bacteria and bioactive peptides with antioxidant and anti-hypertensive properties that may have health-promoting effects [3]. Cheese is also widely consumed and has high economic value. Global production was reported at 26.1 million tonnes in 2024, with Türkiye producing 844 thousand tonnes [4].

Rapid industrialisation and urbanisation, coupled with intensive agricultural activities, have led to an increase in pollutants and harmful potentially toxic metals (PTMs) in the soil, water, and air in recent years [5, 6]. In particular, the concentration of persistent and toxic metals in ecosystems is increasing due to factors such as industrial production, mining activities, fossil fuel use, agricultural practices, and electronic waste [7, 8]. Once released into the environment, these metals can persist for extended periods and enter the food chain through plants and water sources [5, 9]. They may then be transferred to humans when dairy animals consume contaminated feed and water, followed by the consumption of dairy products made from the milk of exposed animals [6]. During cheese production, some of the water is removed with the whey, which can result in contaminants such as PTMs becoming more concentrated in the cheese [10]. Milk and dairy products may contribute to PTM exposure in humans. Their inability to be broken down by the body leads to their accumulation in tissues over time, which increases long-term health risks [11].

In the context of food safety, the quantified metals were evaluated as PTMs, as their health relevance is determined by exposure level and concentration rather than by a simple essential–toxic distinction. PTMs such as iron (Fe), zinc (Zn), copper (Cu), and selenium (Se) are considered essential because they play critical roles in oxygen metabolism, the immune system, enzymatic reactions, prevention of oxidative damage, and metabolic processes within the organism [12]. However, while some PTMs such as chromium (Cr) and nickel (Ni) have beneficial effects in trace amounts for carbohydrate, fat, and protein metabolism and enzymatic activity, they can exhibit toxic effects in high concentrations [13, 14]. In contrast, PTMs such as lead (Pb), cadmium (Cd), and arsenic (As) have no known physiological function in the human body and can produce toxic effects even at low concentrations [15]. Pb has been reported to inhibit brain and nervous system development in children [16], Cd has been associated with carcinogenic effects [17] and immune system disorders [18], and As has been linked to nervous [19], respiratory, and cardiovascular system disorders [20]. Aluminium (Al), on the other hand, is not considered an essential PTM and is associated with neurotoxic effects, particularly exposure to which can lead to neurodegenerative diseases such as Alzheimer’s and Parkinson’s [21, 22].

Cheese is one of the most widely consumed dairy products in Türkiye and is produced under diverse regional, technological, and raw-material conditions. This diversity may influence contaminant occurrence, since PTM contamination can reflect environmental exposure as well as differences in milk source, ripening, and production practices. Although several studies have reported metal concentrations in individual cheese types, comprehensive data comparing products from different regions of Türkiye according to milk source, ripening stage, and production type remain limited. Therefore, a broader assessment is needed to characterise variability and to clarify dietary exposure and potential non-carcinogenic health risk. The aim of this study was to determine PTM levels in cheeses produced in different regions of Türkiye, compare them according to milk source, ripening stage, and production type, and evaluate the associated dietary exposure and non-carcinogenic health risk.

## Materials and Methods

### Materials

A purposive, stratified market-based sampling strategy was used to include commercially available cheeses representing different geographical regions, milk sources, ripening stages, and production types. The sampling was designed for comparative evaluation across these predefined categories rather than for estimating national prevalence. In this study, a total of 84 cheese samples produced in different regions of Türkiye were examined. Two units of each sample were obtained in their original packaging, and the samples were stored in a refrigerator until analysis. The samples were selected to represent the seven geographical regions of Türkiye, and their regional distribution was as follows: East Anatolia Region (EAR, *n* = 14), Marmara Region (MR, *n* = 14), Aegean Region (AR, *n* = 10), Mediterranean Region (MER, *n* = 10), Black Sea Region (BSR, *n* = 11), Central Anatolia Region (CAR, *n* = 14), and Southeast Anatolia Region (SAR, *n* = 11). The samples were also classified according to the type of milk from which they were produced. In this context, the groups were formed as cow milk (*n* = 26), sheep milk (*n* = 15), goat milk (*n* = 17), buffalo milk (*n* = 10), and mixed milk (cow+sheep+goat+buffalo) (*n* = 16). According to ripening status, the samples consisted of ripened (*n* = 45) and fresh (*n* = 39) cheeses; according to production type, they consisted of industrial (*n* = 39) and traditional (*n* = 45) cheeses. This sampling approach allowed PTMs in cheeses to be comparatively evaluated according to regional origin, milk type, ripening status, and production type.

### Sample Preparation and Analysis

PTMs were determined with minor modifications based on the method described by Basaran and Sadighara (2025). Briefly, 0.6 g of each wet cheese sample was weighed into polytetrafluoroethylene (PTFE) digestion vessels, and 6 mL of 65% HNO_3_ (Merck, Darmstadt, Germany) and 2 mL of 30% H_2_O_2_ (Supelco, Darmstadt, Germany) were added. The samples were then digested in a microwave digestion system (Milestone START D, Italy). The digestion program was set to raise the temperature to 200 °C within 15 min, followed by a 15 min holding period at 200 °C under 120 W per vessel, until clear solutions free of visible residue were obtained. After digestion, the solutions were diluted to a final volume of 50 mL with ultrapure water (Millipore Direct Q 8UV, Massachusetts, USA) and stored at 4 °C until analysis.

PTM analysis was performed using an inductively coupled plasma mass spectrometry (ICP-MS) system (Agilent Technologies, 7700 Series, Japan). Instrument operating conditions were as follows: argon was used as the carrier and plasma gas, and helium was used as the collision gas. The argon gas pressure was 500–700 kPa, the helium gas pressure was 90–130 kPa, the carrier gas flow rate was 0.99 L/min, the helium gas flow rate was 4.5 mL/min, and the plasma gas flow rate was 15 L/min. The plasma power was set at 1550 W. The cooling water temperature ranged from 15 to 45 °C, and the spray chamber temperature was maintained at 2 °C. The turbomolecular pump (TMP) speed was 95–100%. Analyses were performed in He/No Gas tune mode under Low Matrix plasma mode. The nebulizer pump speed was 0.3 rps, sample uptake time was 42 s, stabilization time was 45 s, and the integration time was 0.1–1 s per point. Data acquisition and processing were carried out using MassHunter software (version B.01.03).

### Quality Control

External calibration was performed using multi-point calibration curves constructed from certified stock solutions at different concentrations. The analytical performance characteristics of the method, including mean recovery (%), linear calibration equations, coefficients of determination (R^2^), and the limits of detection (LOD) and quantification (LOQ), are summarized in Table S1. Recoveries ranged between 95.5% and 103%, and calibration linearity was high for all analytes (R^2^ = 0.9992–1.0000). The relative standard deviation (RSD) values ranged from 2.22% to 4.57% for all analytes (Table S1).

### Health Risk Assessment

In this study, potential adverse effects associated with PTM exposure through cheese consumption were evaluated using Estimated Daily Intake (EDI), toxicological contribution ratio, Target Hazard Quotient (THQ), and Hazard Index (HI) (Table 1).

**Table 1:** Health risk assessment formula, variables and criteria

Cutoff point	Formula	Variables	Criteria
Estimated Daily Intake (EDI) (µg/kg/day)^a^	$EDI=\frac{C\times IR}{bw\times1000}$	where C represents the concentration of each PTM in the cheese sample (µg/kg), IR is the cheese intake rate (g/day), bw is the body weight (kg), and 1000 is the conversion factor. A cheese consumption rate of 40 g/day for the general population aged > 15 years was used in the calculations [23], while body weight was assumed to be 70 kg.	
Toxicological contribution (TC)^a^ (%)	$TC=\frac{EDI\times100}{TDI\;or\;RDA}$	where TDI is the tolerable daily intake and RDA is the recommended dietary allowance.	PTM exposure levels should remain below the TDI or RDA values. Al: 286 µg/kg/day^1,b, *^ Cr: Female: 0.36 µg/kg/day, Male: 0.50 µg/kg/day^2,c, *^ Fe: Female: 257 µg/kg/day, Male: 114 µg/kg/day^2,c, *^ Ni: 13 µg/kg/day^1,d, *^ Cu: 13 µg/kg/day^2,c, *^ Zn: Female: 114 µg/kg/day, Male: 157 µg/kg/day^2,c, *^ Se: 0.79 µg/kg/day^2,c, *^ Cd: 1 µg/kg/day^2,e, *^ No TDI/RDA values are defined for As and Pb.
Target Hazard Quotient (THQ)^a^ (It has no units)	$$\begin{array}{c}THQ=\\\frac{EDI}{RfD\;or\;TDI\;}\end{array}$$	where RfD/TDI is the oral reference dose/tolerable daily intake (µg/bw/day). RfD for Al, Cr, Fe, Ni, Cu, Zn, As, Se, Cd and Pb 286, 3, 700, 20, 40, 300, 0.3, 5, 1 and 4 µg/kg/day, respectively [24, 25].	THQ/HI value > 1 indicates a potential non-carcinogenic health concern, whereas a THQ/HI value < 1 suggests that the associated health risk is negligible [26].
Hazard Index (HI)^a^ (It has no units)	$$\begin{array}{c}HI=\\\sum_{i=1}^{10}THQ\end{array}$$		

^a^ [27], ^b^ [28], ^c^ [29], ^d^ [30], ^e^ [31]. ^1^ TDI, ^2^ RDA. ^*^For determining TDI/RDA values for PTMs, the weight of an adult individual was considered to be 70 kg

### Statistical Analysis

The data obtained in the study were analyzed using IBM SPSS Statistics version 26. Descriptive statistics were expressed as mean ± standard deviation, median, minimum, and maximum. The assumption of normality was assessed using the Kolmogorov–Smirnov test. Since the data did not meet the assumptions required for parametric analysis, comparisons between independent groups were performed using the Kruskal–Wallis test. Bonferroni-adjusted post hoc comparisons were used to identify statistically significant differences among groups. For the calculation of dietary exposure and risk assessment, values below the LOQ were treated as 0. This approach was used to provide a conservative lower-bound estimate of exposure and risk, particularly because several PTMs showed high < LOQ frequencies. Mean exposure levels were calculated accordingly. Different letters within the same group indicate statistically significant differences (*p* < 0.05). Figures were generated only for statistically significant PTMs.

## Results

### PTM Levels in Cheeses

PTM levels in the different cheese types examined showed wide variability. The concentration ranges for Al, Fe, and Zn were determined as < 0.00215 (LOQ)–7.29, < 0.00769 (LOQ)–38.8, and 0.68–56.6 mg/kg wet weight, respectively. The corresponding ranges for Cr, Ni, Cu, Se, Cd, and Pb were < 0.58 (LOQ)–244, < 1.26 (LOQ)–369, < 2.28 (LOQ)–4067, < 0.91 (LOQ)–1378, < 0.06 (LOQ)–12.3, and < 2.19 (LOQ)–1754 µg/kg wet weight, respectively. In addition, the high < LOQ rates, particularly for As (100%), Se (95.2%), Cd (92.9%), Pb (91.7%), Cr (84.5%), and Ni (66.7%), indicate that these PTMs remained at low or undetectable levels in the majority of the samples (Table 2).

**Table 2:** Some studies reporting PTM levels in cheeses

Cheese type	Number of samples	Country	Milk type	Ripening type	Production type	Al^a^	Cr^b^	Fe^a^	Ni^b^	Cu^b^	Zn^a^	As^b^	Se^b^	Cd^b^	Pb^b^	Reference
Various cheeses	84	Türkiye	Cow milk Sheep milk Goat milk Buffalo milk Mixed	Ripened Fresh	Industrial Artisanal	Min:< LOQ Max: 7.29	Min:< LOQ Max:244	Min: < LOQ Max: 38.8	Min: < LOQ Max: 369	Min: < LOQ Max: 4067	Min: 0.68 Max: 56.6	< LOQ	Min: < LOQ Max:1378	Min: < LOQ Max:12.3	Min:< LOQ Max:1754	This study
Hard goat cheese	30	Ukraine	Goat Milk	Ripened	Artisanal	Min: 0.01 Max: 0.04 Mean: 0.03	Min: 0.58 Max: 1.56 Mean: 1.20	Min: 0.031 Max: 0.07 Mean: 0.05	Min: 0.79 Max: 4.28 Mean: 2.46	Min:3.27 Max:15.6 Mean:7.72	Min: 0.28 Max: 0.59 Mean: 0.48	< LOD	< LOD	Min: 0.16 Max: 0.22 Mean: 0.19	Min: 1.84 Max: 2.52 Mean: 2.28	[32]
Buffalo and cow cheeses	80	Romania	Buffalo milk Cow milk	NS	Industrial Artisanal	Min: 0.02 Max: 0.04 Mean: 0.03	Min: 12.0 Max: 23.0 Mean: 117	N/A	Min: 9.00 Max: 23.0 Mean: 15.6	Min: 160 Max: 280 Mean: 213	Min: 5.65 Max: 8.10 Mean: 6.67	Min: 0.70 Max: 1.20 Mean: 0.98	N/A	< LOD	Min: 5.00 Max: 14.0 Mean: 8.15	[33]
Aho, golot, telli cheeses	30	Türkiye	NS	NS	Artisanal	Min: 0.70 Max: 34.6 Mean: 9.70	Min.17.4 Max: 342 Mean: 77.1	Min: 0.93 Max: 15.6 Mean: 4.96	Min: < LOD Max: 3.68 Mean: 691	Min: 222 Max: 8374 Mean: 759	Min: 6.01 Max: 76.6 Mean: 35.7	Min: < LOD Max: 8.90 Mean: 3.59	N/A	Min: < LOD Max: 10.7 Mean:2.98	Min: 10.6 Max: 1789 Mean: 116	[14]
Feta, Low-salt soft, Istanbolly, Kareish cheeses	100	Egypt	NS	Various	Artisanal	N/A	< LOD	Min: 0.01 Max: 0.08 Mean: 0.04	N/A	Min: 0.29 Max: 2.73	Min: 0.02 Max: 0.54 Mean: 0.34	N/A	Min: 0.11 Max: 0.94 Mean: 0.33	< LOD	< LOD	[34]
Soft, semi-hard, hard cheeses	27	Iraq	NS	NS	Industrial	N/A	N/A	N/A	N/A	Min:< LOD Max: 2130 Mean: 660	N/A	Min: < LOD Max: 210 Mean: 34.0	N/A	Min: < LOD Max: 90.0 Mean: 10.6	Min:< LOD Max: 80.0 Mean: 16.1	[35]
Hard, semi-hard, soft, spread, whey cheeses	102	Greece	Cow milk Sheep milk Goat milk Mixed	NS	Industrial	Min:< LOD Max: 30.0	Min: 600 Max: 600	Min: 1.80 Max: 79.0	N/A	Min: 540 Max: 5000	Min: 3.95 Max: 55.0	Min: < LOD Max: 16.0	Min:< LOD Max: 25.0	N/A	Min:< LOD Max: 160	[12]
Various cheeses	51	Türkiye	NS	Various	Industrial	Min:< LOD Max: 8.01 Mean: 1.75	Min:< LOD Max: 312 Mean: 69.0	N/A	Min: < LOD Max: 461 Mean: 131	Min: 38.7 Max: 469 Mean: 247	N/A	Min: < LOD Max: 1494 Mean: 59.0	N/A	Min: < LOD Max: 0.06 Mean: 0.01	Min: < LOD Max: 0.38 Mean: 0.05	[36]
Sheep cheese	12	Slovakia	Sheep milk	Fresh	Artisanal	Mean: 3.35 ± 2.25	Mean: 40.0 ± 10.0	Mean: 6.57 ± 0.84	Mean: 700 ± 520	Mean: 260 ± 80	Mean: 14.3 ± 5.60	Mean: 730 ± 290	Mean: 380 ± 350	< LOD	Mean: 130 ± 90	[37]
Sheep cheese	144	Romania	Sheep milk	Fresh	Artisanal	N/A	< LOD	N/A	< LOD	Min: 310 Max: 4020 Mean: 1080–2920	Min: 1.11 Max: 59.8 Mean: 1.82–38.4	< LOD	N/A	Min: < LOD Max: 120 Mean: 30.0–60.0	Min: < LOD Max: 760 Mean: 170–580	[38]
Various cheeses	80	Brazil	NS	NS	Artisanal	N/A	Min: 20.8 Max: 147 Mean: 77.7	N/A	N/A	Min: 91.3 Max: 964 Mean 560	N/A	Min: < LOD Max: NS Mean: 27.2	N/A	Min: 5.25 Max: 27.2 Mean: 14.8	Min: 5.78 Max: 274 Mean: 96.4	[39]
Various cheeses	88	Mexico	NS	Various	Artisanal	N/A	N/A	Min: 55.8 Max: 97.4 Mean: 63.8	Min: 0.20 Max 13.2 Mean: 3.50	Min: 0.70 Max: 43.7 Mean: 20.1	Min: 0.02 Max: 0.44 Mean: 0.12	N/A	N/A	Min: < LOD Max: 15.6 Mean: 2.30	Min: 0.30 Max: 12.5 Mean: 4.90	[40]
Goat cheese	31	Slovakia	Goat milk	Various	Artisanal	Min:< LOD Max: 13.9	< LOD	Min: 2.05 Max: 4.84	< LOD	Min. 470 Max: 990	Min: 18.6 Max: 30.7	< LOD	< LOD	< LOD	< LOD	[41]
Coalho cheese	16	Brazil	Goat milk	Fresh	Artisanal	< LOD	< LOD	< LOD	< LOD	Min: 420 Max. 580	Min: 20.3 Max: 23.4	N/A	N/A	< LOD	< LOD	[42]
Galician cheese	30	Spain	Cow milk	NS	Industrial	N/A	Mean: 97.4	Mean: 4.18	Mean: 48.6	Mean: 516	Mean: 56.7	Mean: 4.31	Mean: 529	Mean: 2.21	Mean: 12.2	[43]
Various cheeses	128	Bangladesh	NS	NS	Industrial	N/A	Min: 76.0 Max: 886	Min: 0.17 Max: 0.59	N/A	Min: 118 Max: 2312	Min: 0.09 Max: 0.20	Min: 5.00 Max: 88.0	N/A	Min: 16.0 Max: 42.0	Min: 5.00 Max: 25.0	[44]
Imeruli Cheese	16	Georgia	Cow milk	NS	Artisanal	N/A	Mean: 35.0 ± 17.0	Mean: 69.1 ± 64.9	Mean: 11.0 ± 7.00	Mean: 1261 ± 739	Mean: 75.9 ± 52.5	N/A	Mean: 1003 ± 901	Mean: 2.00 ± 1.50	Mean: 121 ± 93.0	[45]
Sulguni cheese	9	Georgia	Cow milk	NS	Artisanal	N/A	Mean: 79.0 ± 57.0	Mean: 101 ± 91.2	Mean: 26.0 ± 29.0	Mean: 2463 ± 2314	Mean: 125 ± 97.8	N/A	Mean: 3060 ± 3144	Mean: 7.00 ± 3.00	Mean: 258 ± 215	
Various cheeses	30	Sudan	Cow milk	NS	Artisanal	Min: 22.0 Max: 153	Min: 100 Max: 300	Min: 40.0 Max: 116	< LOD	< LOD	Min: 18.0 Max: 28.0	< LOD	Min: 200 Max: 300	N/A	N/A	[46]

For this study, ^a^ indicates mg/kg wet weight and ^b^ indicates µg/kg wet weight

**Fig. 1:**
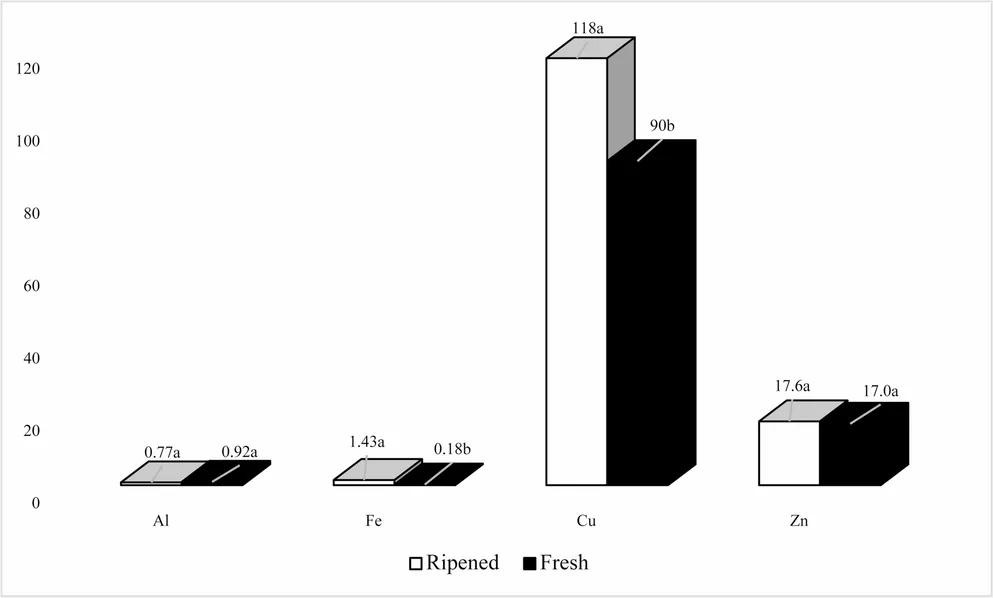
Statistical comparison of PTM levels (median) in cheeses classified by ripening stage. Al, Fe, and Zn: mg/kg wet weight; Cu: µg/kg wet weight

Ripened cheeses showed the maximum Al and Cd concentrations, while fresh cheeses had the highest values for Cr, Fe, Ni, Cu, Zn, Se, and Pb (Table S2). In the ripening-stage comparison, Fe was higher in ripened samples, whereas Cu was higher in fresh samples (*p* < 0.05); no significant difference was observed for Al or Zn (*p* > 0.05) (Fig. 1).

**Fig. 2:**
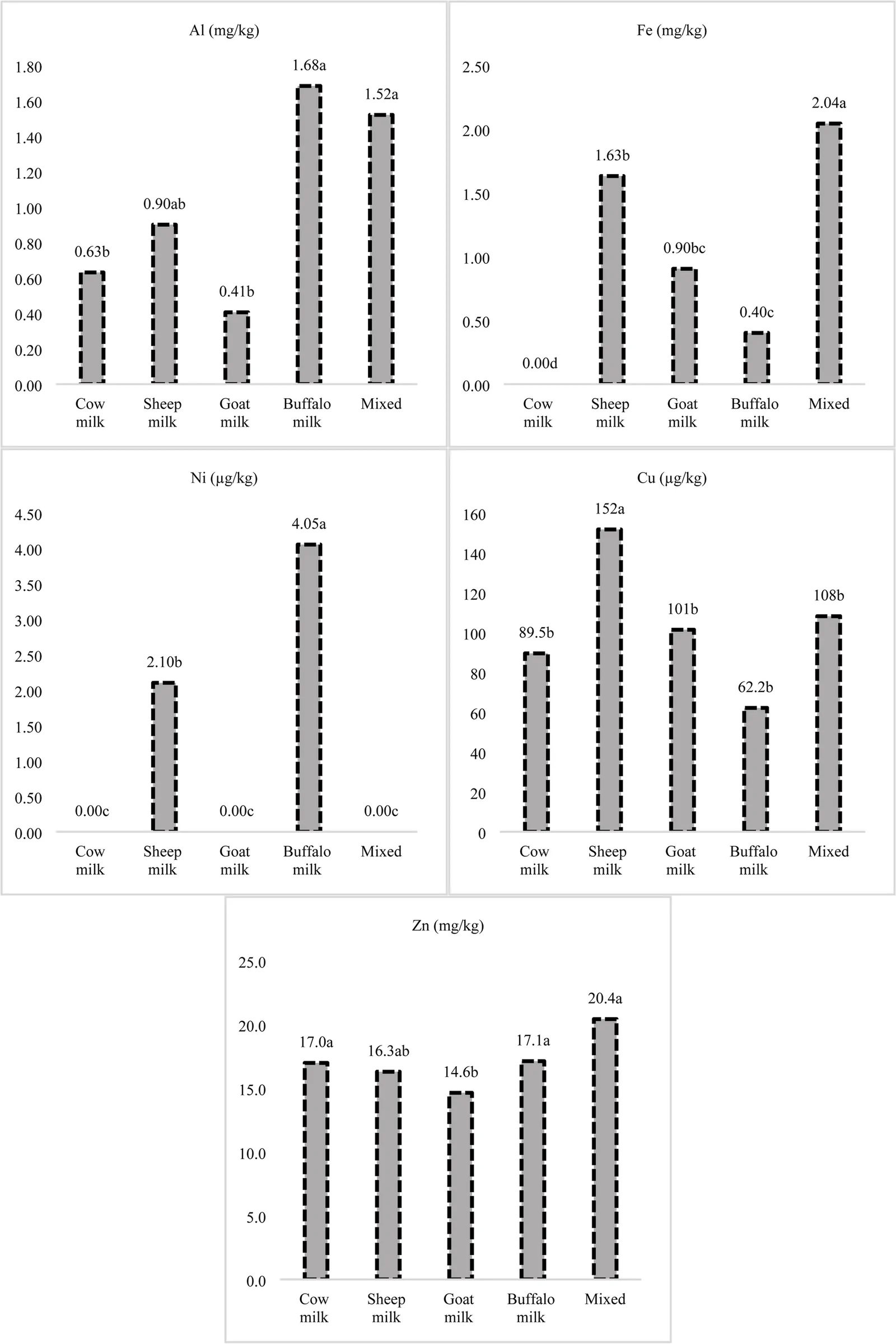
Statistical comparison of PTM levels (median, wet weight) in cheeses classified by animal species used for milk production

Maximum Al and Cu levels were identified in cheeses produced from cow milk, whereas the highest Cr and Zn levels were found in sheep and goat milk cheeses, respectively. The highest Fe and Ni values were determined in cheeses produced from mixed milk. Although Se, Cd, and Pb were determined as < LOQ in most samples, they reached higher maximum values in cheeses produced from the milk of certain animal species (Se: goat milk [1378 µg/kg wet weight]; Cd: mixed milk [12.3 µg/kg wet weight], sheep milk [8.69 µg/kg wet weight], and buffalo milk [8.69 µg/kg wet weight]; Pb: cow milk [1754 µg/kg wet weight] and goat milk [1281 µg/kg wet weight]) (Table S3). Statistical evaluation revealed that the PTM profile of the cheese samples varied according to the animal species from which the milk was obtained (*p* < 0.05). Cheeses produced from buffalo milk were particularly prominent in terms of Al and Ni, whereas cheeses produced from mixed milk showed higher levels of Fe and Zn. Cheeses produced from sheep milk were distinguished from the other groups by their Cu levels and also belonged to the high group in terms of Fe. In contrast, cheeses produced from cow milk showed the lowest level particularly for Fe, whereas cheeses produced from goat milk had lower levels in terms of Al and Zn (Fig. 2).

**Fig. 3:**
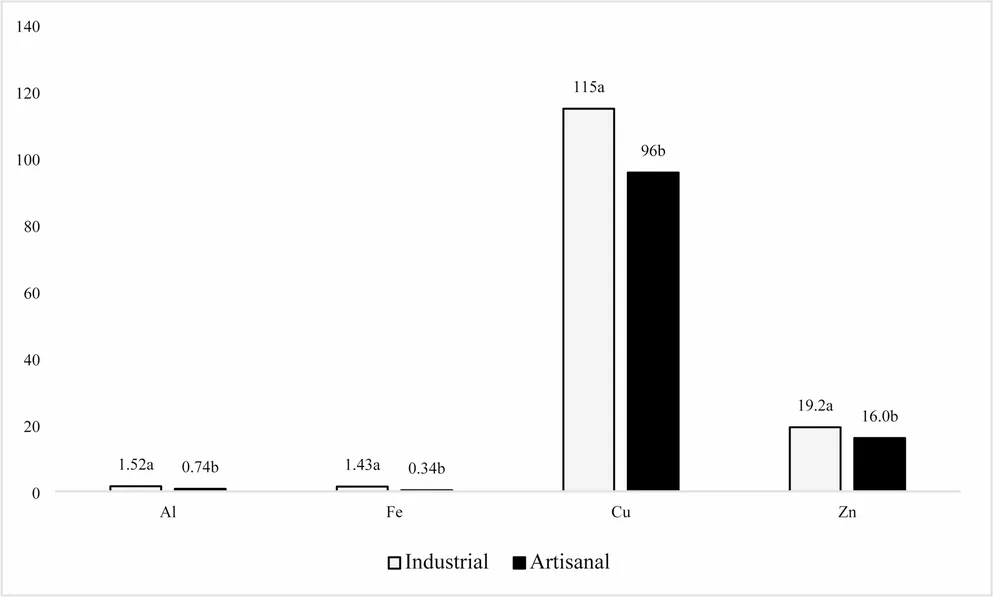
Statistical comparison of PTM levels (median) in cheeses classified by production type. Al, Fe, and Zn: mg/kg wet weight; Cu: µg/kg wet weight

Maximum Al, Cr, Fe, Ni, and Cd levels were identified in industrial cheeses, whereas the highest Cu, Zn, Se, and Pb values were found in traditional cheeses (Table S4). Levels of Al, Fe, Cu, and Zn were higher in industrial cheeses than in traditional cheeses (*p* < 0.05) (Fig. 3).

**Fig. 4:**
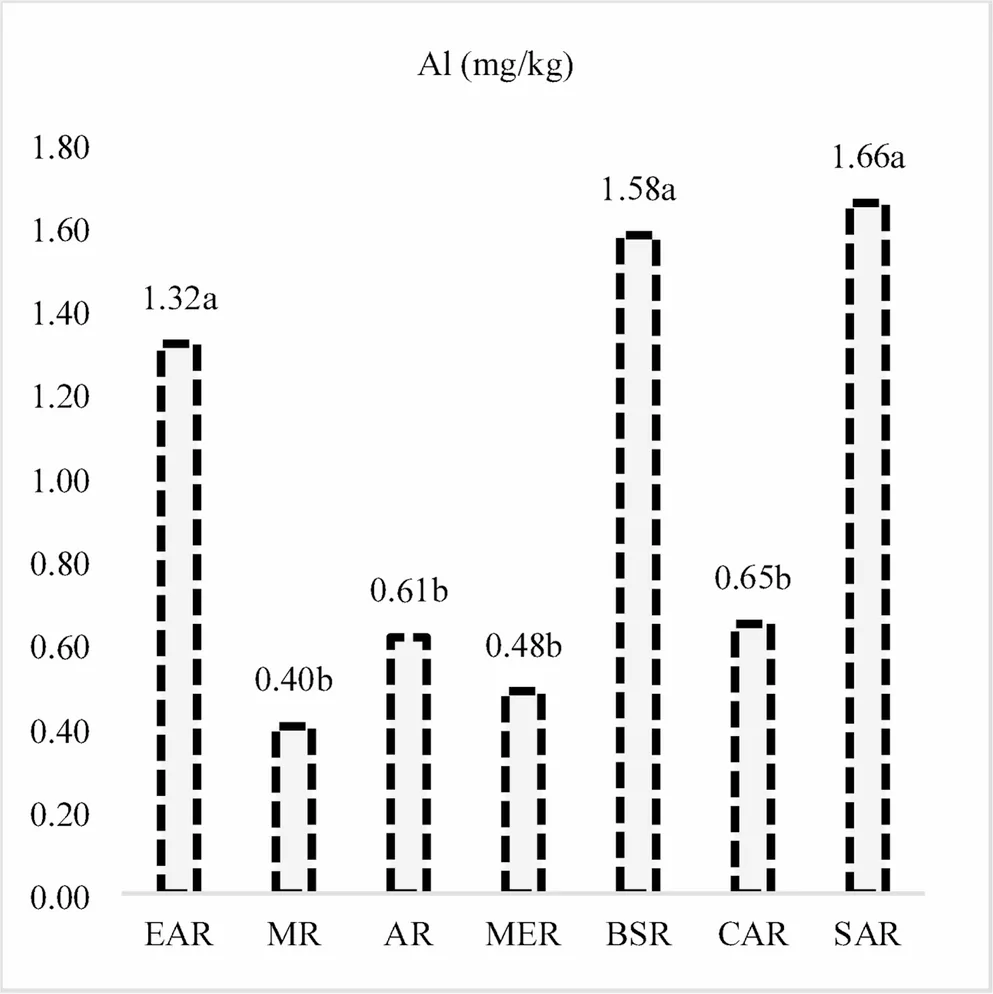
Statistical comparison of PTM levels (median, wet weight) in cheeses classified by region. EAR: East Anatolia Region, MR: Marmara Region, AR: Aegean Region, MER: Mediterranean Region, BSR: Black Sea Region, CAR: Central Anatolia Region, SAR: Southeast Anatolia Region

PTM levels in the analyzed cheese samples showed wide variability by region. Maximum Al and Zn levels were identified in CAR, the maximum Cr level in AR, and the maximum Cu level in BSR, whereas the highest Fe value was detected in MR and the highest Ni value in EAR. Although Se, Cd, and Pb were determined at < LOQ in most samples, they reached higher maximum values in some regions (Se: EAR [1378 µg/kg wet weight] and SAR [730 µg/kg wet weight]; Cd: MR [12.3 µg/kg wet weight]; Pb: BSR [1754 µg/kg wet weight] and MR [93.5 µg/kg wet weight]) (Table S5). Regional comparisons showed significant differences in Al, Fe, Zn, Ni, and Cu (*p* < 0.05). Al was more elevated in samples from EAR, BSR, and SAR, but lower in MR, AR, MER, and CAR. In terms of Fe, the highest levels were identified in MR and SAR, whereas the lowest level was determined in BSR. The distribution of Zn also showed regional differences; MR and BSR had higher levels, whereas AR, MER, and CAR had lower levels (Fig. 4).

### Dietary Exposure

The highest mean exposure value was observed for Zn (10.3 µg/kg/day), followed by Fe (1.07 µg/kg/day) and Al (0.77 µg/kg/day). In contrast, lower mean exposure values were observed for Cu (0.13 µg/kg/day), Se (0.02 µg/kg/day), Pb (0.02 µg/kg/day), and Ni (0.01 µg/kg/day). Mean exposure for Cr and Cd remained at negligible levels, whereas the exposure value for As was calculated as 0.00 µg/kg/day because it was not detected in the cheese samples. Maximum exposure values showed a similar distribution; the highest values were determined for Zn (32.3 µg/kg/day) and Fe (22.2 µg/kg/day), followed by Al (4.17 µg/kg/day) and Cu (2.32 µg/kg/day). The low maximum exposure observed for the other PTMs (Pb: 1.00 µg/kg/day; Se: 0.79 µg/kg/day; Ni: 0.21 µg/kg/day; Cr: 0.14 µg/kg/day; Cd: 0.01 µg/kg/day) indicates that cheese consumption represents a limited source of exposure for these PTMs. Exposure levels according to cheese ripening stage, the animal species from which the milk was obtained, production type, and region are presented in Tables S6–S9.

When all cheese samples were evaluated together, the mean contribution rate was calculated as 0.27% for Al, 0.07% for Ni, 1.00% for Cu, 2.06% for Se, and 0.02% for Cd. For the PTMs for which different reference values were used according to sex, the mean contribution rate was calculated as 0.02% for women and 0.01% for men for Cr, 0.42% for women and 0.94% for men for Fe, and 6.55% for women and 9.02% for men for Zn (Table S10).

### Non-carcinogenic Risk Assessment

The mean (min–max) THQ values of all cheese samples were calculated as 0.01 ± 0.01 (0.00–0.03), < 0.00 ± 0.01 (0.00–0.05), < 0.00 ± 0.00 (0.00–0.03), < 0.00 ± 0.00 (0.00–0.01), < 0.00 ± 0.01 (0.00–0.06), < 0.03 ± 0.02 (0.00–0.11), < 0.00 ± 0.02 (0.00–0.16), < 0.00 ± 0.00 (0.00–0.01), and 0.01 ± 0.03 (0.00–0.25) for Al, Cr, Fe, Ni, Cu, Zn, Se, Cd, and Pb, respectively. Among the PTMs, the highest contribution in terms of mean THQ was observed for Zn, followed by Al and Pb. When maximum values were taken into account, the highest THQ value was determined for Pb (0.25), followed by Se (0.16) and Zn (0.11). THQ levels according to the ripening stage of the cheese, the animal species from which the milk was obtained, production type, and region are presented in Tables S11–S14.

**Fig. 5:**
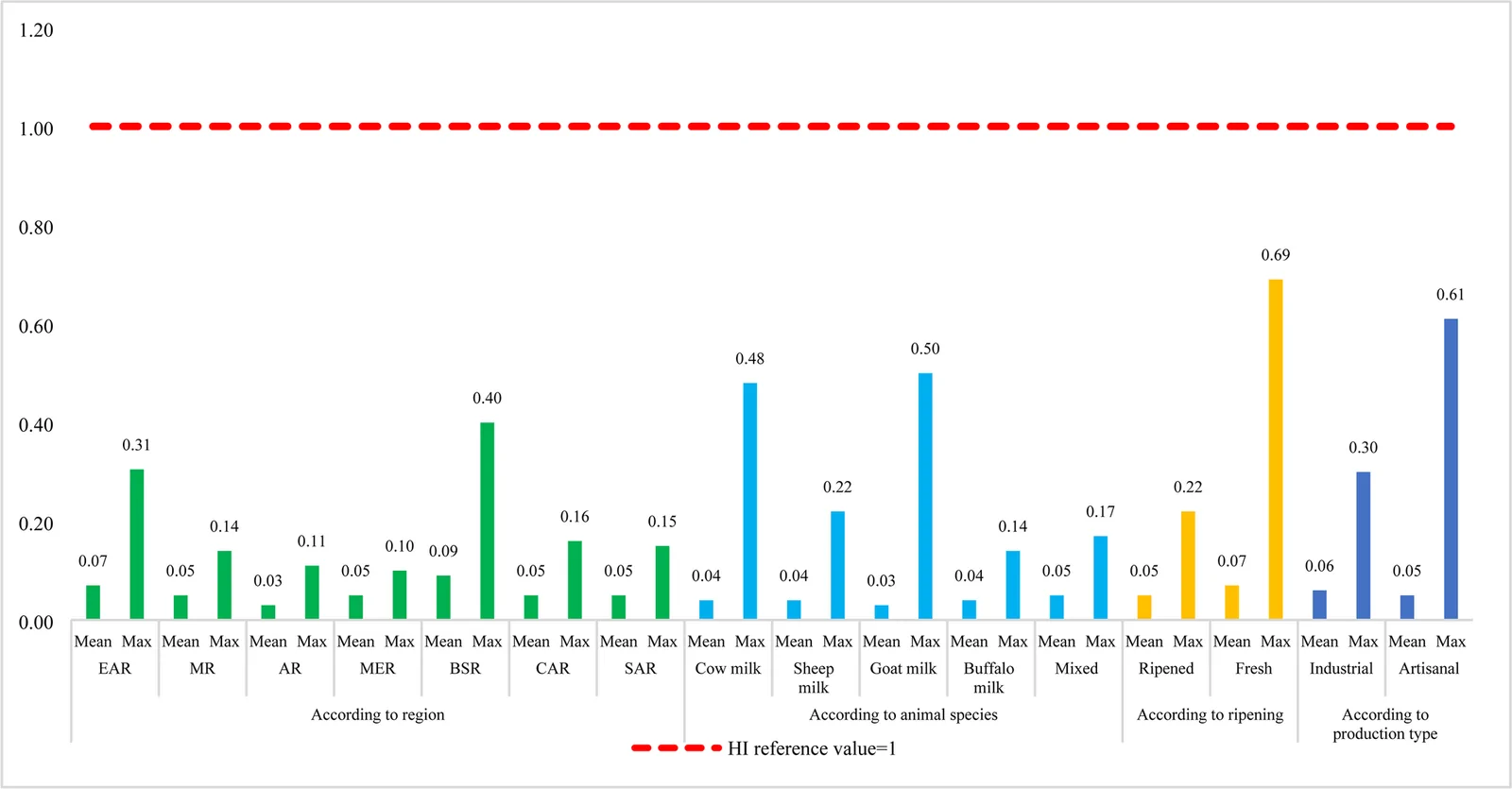
HI values of cheeses classified by region, animal species, ripening stage, and production type

The HI value, which represents the overall non-carcinogenic risk associated with PTM exposure through cheese consumption, was calculated as 0.06 ± 0.09. Nevertheless, the fact that HI values remained below the threshold value in all subgroups indicates that the observed differences were relative in magnitude and that cheese consumption generally exhibited a low overall non-carcinogenic risk profile (Fig. 5).

## Discussion

### PTM Levels in Cheeses

Regulatory limits for milk and dairy products are available only for a limited number of PTMs. In the present context, maximum limits are defined as 300 µg/kg for Cr, 100 µg/kg for As, and 20 µg/kg for Pb [47, 48], whereas no specific regulatory limit has been established for Al, Fe, Ni, Cu, Zn, Se, or Cd. When these limits were taken into account, all analyzed cheese samples were within the acceptable range for Cr and As, whereas four samples exceeded the legal limit established for Pb. In a recent study conducted in Türkiye, only one of the 51 cheese samples exceeded the legal limit for Cr, Pb levels were within the regulatory limits in all samples, whereas As levels exceeded the legal limit in five samples [36]. The Pb exceedances should be interpreted separately from the overall non-carcinogenic risk estimates. Although THQ and HI values remained below 1 under the evaluated exposure scenario, regulatory exceedance indicates a product-level food safety concern. Therefore, the low overall risk estimates do not eliminate the need for monitoring individual cheese samples, particularly because Pb may occur sporadically and may not be adequately represented by mean or median values alone.

When the present findings are evaluated together with the literature, they appear to show partial agreement with previously reported cheese data, although a wider variability is evident for some PTMs. The Al results showed a broader distribution than the recent data reported from Türkiye and the narrower ranges documented in Romania [33, 36]. Data reported for Aho, Golot, and Telli cheeses in Türkiye also indicate wide variability in terms of Al, Cr, Fe, Ni, Cu, Zn, As, Cd, and Pb, with particularly higher maximum levels reported for Al, Cr, Cu, Zn, and Pb [14]. In contrast, Fe levels remain below the higher maximum levels reported from Mexico and Sudan [40, 46]. While Ni levels show a wider variability than the 9.00–23.0 µg/kg range reported from Romania [33], the present study reveals a more remarkable profile for Cu and Zn. In particular, the maximum levels determined for Cu are higher than the ranges reported in most of the selected studies, whereas Zn levels are above the 5.65–8.10 mg/kg range reported from Romania and the minimum levels reported from Mexico and Bangladesh [33, 40, 44]. The absence of detectable As in the analyzed cheese samples differs from the < LOQ–1494 µg/kg range reported from Türkiye, the 0.70–1.20 µg/kg levels reported from Romania, and the measurable As presence reported from Bangladesh, while it is consistent with the studies reported from Sudan [33, 36, 44, 46]. For Se, the maximum level observed in the present study is considerably higher than the ranges of 0.11–0.94 µg/kg reported from Egypt and < LOD–25.0 µg/kg reported from Greece [12, 34]. The levels of Cd generally remained low, while the maximum Pb values were consistent with those reported by Erol and Ürkek [14] but exceeded the ranges reported in many other selected studies [12, 33, 34, 36] (Table 2). Maximum values were considered in the discussion because PTM contamination was highly heterogeneous and igh concentrations may be relevant for food safety surveillance, even when average or median values remain low.

The differences observed among studies arise not only from variation in PTM levels themselves, but also from differences in sampling strategy, cheese type, milk source, production conditions, and analytical approach. Differences in animal species, ripening status, production scale, and processing steps may directly affect the composition of the cheese matrix and, consequently, PTM accumulation. In addition, factors such as the feed and water sources of the animals, regional environmental exposure, geological structure, industrial activities, and mining operations create considerable variability at the raw material level. During the production chain, stages such as equipment contact, salting, storage, and packaging may also provide additional contributions for certain PTMs. Furthermore, differences in result reporting, such as the use of min–max, mean, or mean ± SD values and different LOD/LOQ thresholds, further limit direct comparability.

For ripened cheeses, the present findings indicate a wider variability when compared with the study conducted in Ukraine. While Davydovych et al. [32] reported narrower and lower ranges for Al, Cr, Fe, Ni, Cu, Zn, Cd, and Pb in ripened cheeses, the ripened cheeses in the present study showed higher maximum levels for most of these PTMs; in contrast, As was not detected in either study. In terms of fresh cheeses, the present findings again present a more heterogeneous picture when compared with the studies conducted in Slovakia and Brazil [37, 42]. In particular, while measurable As and Se were reported in Almášiová et al. [37], As was not detected in the present study; in contrast, a wider distribution was observed for Se, Cu, Zn, and Pb. Similarly, while Al, Cr, Fe, and Ni were reported at undetectable levels in fresh cheeses in Souza Silva et al. [42], measurable maximum levels were determined for these PTMs in the present study; in addition, higher upper values were also observed for Cu and Zn. These comparisons suggest that the PTM profile, particularly in the fresh cheese group, exhibits a wider variation compared with the cheese samples reported in the literature (Table 2).

When evaluated according to the animal species from which the milk was obtained, the Fe, Ni, Cu, and Se levels determined in cheeses produced from cow milk appear generally comparable with the data reported from Spain, although they remain below the higher Fe, Cu, and Se levels reported from Georgia. In contrast, higher maximum levels are noteworthy in terms of Pb [43, 45]. In cheeses produced from sheep milk, Zn levels appear to be consistent with the range reported from Romania, whereas Cu, Cd, and Pb levels remain below the maximum levels reported in the same study [38]. Cheeses produced from goat milk, on the other hand, exhibit a broader PTMs profile, particularly when compared with the data reported from studies conducted in Slovakia and Ukraine. Indeed, the maximum levels determined for Al, Cr, Ni, Zn, Se, and Pb exceed the ranges reported by Almášiová et al. [41] and Davydovych et al. [32], whereas the non-detection of As is consistent with these studies. Although directly comparable data are more limited for cheeses produced from buffalo milk, when the values reported in the study conducted on buffalo and cow cheeses in Romania are evaluated together, a wider variability is noteworthy in the present study, particularly in terms of Al, Ni, Zn, and Pb [33]. As directly equivalent literature is limited for the mixed milk group, it can only be said that this group may be evaluated at a general level together with the broad ranges reported from Greece for multiple milk sources [12]. Overall, this comparison suggests that there is a more pronounced heterogeneity in some PTMs, particularly in cheeses produced from goat, buffalo, and cow milk, whereas cheeses produced from sheep milk exhibit a distribution closer to the selected literature (Table 2).

When evaluated by production type, the present findings do not indicate a uniform distinction between industrial and artisanal cheeses. The higher maximum values of Al, Cr, Fe, Ni, and Cd in the industrial group are consistent with the wide Cr and Ni ranges previously reported for cheeses marketed in Türkiye and with industrial cheese data from Spain and Iraq [35, 36, 43]. By contrast, the elevated Cu, Zn, Se, and Pb maxima in the artisanal group correspond more closely to the broad Cu, Zn, and Pb ranges reported for traditional Turkish cheeses [14]. The narrower ranges reported in Romania for Al, Ni, Cu, Zn, As, and Pb suggest that production-type-related variability was more pronounced in the present study [33].

Overall, PTM distribution appears to reflect a multidimensional structure shaped by different stages of the dairy production chain rather than by a single variable. Ripening status, animal species, production type, and regional environmental conditions each contributed to the observed pattern, but none alone explained the entire variation. Differences among published cheese studies support this interpretation. Industrial activities, agricultural practices, fertiliser and pesticide use, traffic, mining, fossil fuel consumption, and proximity to residential areas can disperse PTMs into the environment [49, 50] and facilitate their transfer into the milk chain through the feed–water–soil route [51].

The present findings are also consistent with this framework. The higher Fe levels found in ripened cheeses may be related to moisture loss occurring during the ripening process and the associated increase in concentration. In contrast, the higher Cu levels observed in fresh cheeses suggest that this PTM may be influenced not only by the ripening process, but also by factors such as the initial milk composition, production conditions, and equipment contact. The absence of a significant difference for Al and Zn indicates that ripening does not exert a similar effect on every PTM. Likewise, the higher PTM levels observed in industrial production may be associated with a more complex processing chain, more intensive equipment contact, and the accumulation of environmental and technological inputs in the same product due to milk collection from different sources. The higher levels of Al and Ni in cheeses obtained from buffalo milk, Cu in sheep cheeses, and Fe and Zn in mixed cheeses indicate that different milk types together with production chains may shape the PTM profile. These differences may be partly explained by the ability of some PTMs to bind to milk casein and the fat phase and pass into the curd [10, 45, 52]. Changes during coagulation, ripening, and cheese-type-specific processing may also affect PTM concentrations [32, 43, 53–56]. The regional results also support this picture, with industrial and population pressure appearing more explanatory in some regions, mining and geological structure in others, and pasture-based livestock production and production systems in others. Therefore, in the future, rather than analyzing cheese samples alone, holistic approaches that evaluate milk, feed, water, soil, and production equipment together will be more appropriate for identifying the sources of the observed differences more robustly.

### Dietary Exposure

Estimated daily intake varied markedly among the analysed PTMs. Compared with previous cheese studies, the exposure estimates obtained here differed considerably [14, 32, 34, 36, 37, 39]. This variation may reflect both differences in metal concentrations and variation in the amount of cheese consumed at individual and geographical levels.

The contribution of exposure arising from cheese consumption to the recommended TDI/RDA levels for the PTMs examined was generally found to be low. A similar pattern was also observed when the subgroups were taken into account; however, some PTMs showed relatively higher contributions in certain classifications. The mean contribution rate was particularly more pronounced for Zn, varying approximately between 3.97% and 10.3% across different subgroups, whereas for Se this rate was found to range between 0.00% and 12.4%. Under the upper-limit scenario, contribution rates increased particularly for Se (up to 99.7%), Zn (20.6–28.4%), Fe (8.63–19.4%), and Cu (17.9%), whereas they remained at lower levels for Al, Cr, Ni, and especially Cd. These results indicate that cheese consumption makes a generally low and limited contribution to the daily toxicological or nutritional reference values for the PTMs examined at mean exposure levels. Nevertheless, under some subgroups and maximum exposure scenarios, the contribution rates for Se, Zn, Fe, and Cu became more pronounced. Since no defined TDI value is available for As and Pb, contribution rates were not calculated for these PTMs (Table S10).

The generally low concentrations of As, Cd, and, except for a limited number of samples, Pb represent a favorable finding, considering that the International Agency for Research on Cancer classifies As and Cd as Group 1 carcinogens, Ni and Pb as Group 2B carcinogens, and Cr as a Group 3 agent [57]. The finding that exposure to PTMs from cheese consumption generally remained below the TDI/RDA is consistent with the literature [36]. However, in the study by Almášiová et al. (2024), the toxicological contribution of PTM exposure was highly scenario-dependent and was particularly pronounced for As, especially in children. The authors reported that As intake exceeded the applicable reference level in some adult scenarios and rose markedly further in child scenarios, while contributions from most other PTMs remained substantially lower. Among nutritionally relevant PTMs, Se and, to a lesser extent, Zn made the largest contributions to recommended intake levels.

### Non-carcinogenic Risk Assessment

THQ values were generally low, and all remained below 1. This indicates a low non-carcinogenic risk under the evaluated exposure scenario and is consistent with many studies from Ukraine, Türkiye, Egypt, Iraq, Brazil, Mexico, and Bangladesh [14, 32, 34–36, 39, 40, 44]. However, THQ values above 1 reported for sheep cheese in Slovakia and various cheeses in Sudan show that risk levels may vary by product type and contamination context [37, 46].

When all samples were evaluated together, HI values ranged between 0.00 and 0.70. HI values remained below 1 in all samples, indicating that combined PTM exposure through cheese consumption was associated with a low non-carcinogenic risk in the samples examined. Subgroup evaluations also supported this overall conclusion. In the regional comparison, the relatively highest total risk contribution was observed in the BSR group, whereas in the classification according to animal species, the mixed milk group stood out in terms of mean level, while the highest individual contributions were observed in cheeses produced from goat and cow milk. According to ripening status, fresh cheeses appeared more noteworthy, whereas according to production type, industrial cheeses were more remarkable at the mean level and traditional cheeses in terms of the highest individual contribution.

Available evidence indicates that the non-carcinogenic health risk associated with cheese consumption is generally low, although this conclusion is not uniform across all studies. Rocha et al. [39] and Yi-Jie Dai et al. [15] reported HI values below 1 for all cheese types, indicating no significant non-carcinogenic risk associated with cheese consumption. Similarly, Erol and Ürkek [14] found that 93.3% of the analyzed cheese samples remained below the critical HI threshold, although some samples exceeded the safe limit, suggesting that the overall risk was low but not negligible in every case. In another study, HI values were generally below the threshold, but HI > 1 was reported for one feta cheese sample due to its high As level [36]. In contrast, Almášiová et al. [37] reported HI > 1 under some consumption scenarios and concluded that the regular consumption of sheep milk and cheese produced in an environmentally burdened area could not be considered safe, particularly for children. In this context, while current findings support the idea that cheese consumption generally has a low non-carcinogenic risk profile, differing results regarding THQ and HI values in the literature highlight the importance of regular monitoring for specific product groups and production environments. The risk assessment should also be interpreted in relation to the exposure assumptions used in this study. THQ and HI values were calculated using a general cheese consumption rate and adult body weight assumption. Therefore, the findings indicate a low non-carcinogenic risk under the evaluated scenario, but they may not fully represent high consumers, children, or populations with different dietary habits. This limitation is particularly relevant for elements such as Pb, where isolated exceedances were observed despite low overall HI values.

## Conclusion

This study evaluated PTM levels in cheeses produced in different regions of Türkiye and examined how these levels varied according to ripening stage, milk source, production type, and geographical origin. Overall, PTM concentrations showed considerable variability among samples, indicating that cheese contamination cannot be explained by a single factor alone. In particular, some PTMs differed significantly according to ripening stage, milk source, production type, and region, while others remained consistently low or below the quantification limit. Among the analyzed PTMs, Zn was the most prominent contributor to overall exposure, whereas As was not detected in any sample. The dietary exposure assessment showed that cheese consumption contributed only a limited proportion to the relevant toxicological or nutritional reference values for most PTMs. Likewise, the calculated THQ and HI values remained below the critical threshold, suggesting a low overall non-carcinogenic health risk under the exposure assumptions used in this study. Nevertheless, the exceedance of the legal limit for Pb in four samples shows that the overall low THQ and HI values should not be interpreted as the absence of contamination concern in all individual products. Therefore, continuous monitoring of PTMs in cheese remains necessary, particularly for products that differ in origin, production practices, and milk source. Future studies integrating cheese, milk, feed, water, soil, and processing equipment would provide a more robust basis for identifying contamination sources and clarifying the mechanisms underlying the observed variability.

## Supplementary Information

Below is the link to the electronic supplementary material.

Supplementary Material 1 (DOCX 1.42 MB)

## Data Availability

Datasets generated and/or analyzed during the current study will be shared with the corresponding authors upon reasonable request.
