# Supplementary material for: Potentially Toxic Metal Levels in Cheeses Produced in Türkiye: Regional Variation, Milk Source, Ripening Stage, Production Type, and Associated Dietary Exposure and Health Risk
Source: Biol Trace Elem Res. 2026 Jun 27;204(10):7426–40. doi: 10.1007/s12011-026-05198-z (PMC13615985; doi:10.1007/s12011-026-05198-z)
Supplement: Supplementary file 1 [file 12011_2026_5198_MOESM1_ESM.docx]

**Potentially toxic metal levels in cheeses produced in Türkiye: Regional variation, milk source, ripening stage, production type, and associated dietary exposure and health risk** Hüseyin Ender Gürmeriç^1,*^, Mustafa Şengül^2^, Burhan Basaran^3,*^

^1,*^Department of Food Processing, Şiran Mustafa Beyaz Vocational School, Gümüşhane University, Şiran, Gümüşhane, 29700, Türkiye.

E-mail address: hegurmeric@gumushane.edu.tr

^2^Department of Food Engineering, Faculty of Agriculture, Atatürk University, Erzurum, 25240, Turkey

E-mail address: msengul@atauni.edu.tr

^3^Department of Nutrition and Dietetics, Faculty of Health Sciences, Recep Tayyip Erdogan University, Rize, 53100, Türkiye.

E-mail address: burhan.basaran@erdogan.edu.tr

* Corresponding Author e-mail: hegurmeric@gumushane.edu.tr

* Corresponding Author e-mail: burhan.basaran@erdogan.edu.tr

Orcid Number of Authors:

Hüseyin Ender Gürmeriç: 0000-0001-8636-1031

Mustafa Şengül: 0000-0001-8447-2256

Burhan Basaran: 0000-0001-6506-6113

**Table S1.** Analysis of the recovery, LOD, LOQ and calibration (R^2^) for the PTMs

| **PTMs** | **Mean Recovery (%)** | **Linear equation x, y (μg/kg)** | **R^2^** | **LOD (µg/kg)** | **LOQ (µg/kg)** | **RSD (%)** |
| --- | --- | --- | --- | --- | --- | --- |
| Al | 101 | y=320085.2703*x+1623.4200 | 0.9992 | 0.64 | 2.15 | 4.38 |
| Cr | 96.5 | y=8036.7101*x+1088.7067 | 0.9998 | 0.17 | 0.58 | 3.99 |
| Cu | 95.6 | y=9013.5625*x+2063.5433 | 0.9997 | 0.56 | 1.88 | 2.28 |
| Fe | 102 | y=6532413.7127*x+10463.3555 | 1.0000 | 2.29 | 7.69 | 2.74 |
| As | 101 | y=1065.1118*x+204.0000 | 0.9998 | 0.29 | 0.96 | 3.54 |
| Se | 97.5 | y=98.7070*x+119.6667 | 0.9999 | 0.27 | 0.91 | 3.46 |
| Ni | 103 | y=3214.8699*x+569.6767 | 1.0000 | 0.38 | 1.26 | 2.22 |
| Pb | 102 | y=39055.0519*x+7128.5033 | 0.9999 | 0.66 | 2.19 | 4.57 |
| Zn | 98.5 | y=1527169.2595*x+6438.0600 | 0.9999 | 0.06 | 0.22 | 4.03 |
| Cd | 95.5 | y=5201.0796*x+378.0067 | 0.9999 | 0.02 | 0.06 | 2.88 |

PTMs: Potentially toxic metals, LOD: Limit of detection, LOQ: Limit of quantification, RSD: Relative Standard Deviation.

**Table S2.** PTM levels in cheeses classified by ripening stage

| **Ripening type** | **Al^a^** | **Cr^b^** | **Fe^a^** | **Ni^b^** | **Cu^b^** | **Zn^a^** | **As^b^** | **Se^b^** | **Cd^b^** | **Pb^b^** |  |
| --- | --- | --- | --- | --- | --- | --- | --- | --- | --- | --- | --- |
| **Ripened** | | | | | | | | | | | |
| <LOD% | - | - | - | - | - | - | - | - | - | - |  |
| <LOQ% | 15.6 | 80.0 | 17.8 | 71.1 | 15.6 | - | 100 | 93.3 | 93.3 | 97.8 |  |
| Min | <LOQ | <LOQ | <LOQ | <LOQ | <LOQ | 0.68 | <LOQ | <LOQ | <LOQ | <LOQ |  |
| Max | 7.29 | 31.3 | 10.8 | 108 | 556 | 33.4 | <LOQ | 730 | 12.3 | 93.5 |  |
| **Fresh** | | | | | | | | | | | |
| <LOD% | - | - | - | - | - | - | - | - | - | - |  |
| <LOQ% | 10.3 | 87.2 | 43.6 | 61.5 | 12.8 | - | 100 | 94.9 | 92.3 | 89.7 |  |
| Min | <LOQ | <LOQ | <LOQ | <LOQ | <LOQ | 5.41 | <LOQ | <LOQ | <LOQ | <LOQ |  |
| Max | 5.89 | 244 | 38.8 | 369 | 4067 | 56.6 | <LOQ | 1378 | 0.90 | 1754 |  |

^a^ mg/kg dry weight, ^b^ µg/kg dry weight, LOD: Limit of detection, LOQ: Limit of quantification.

**Table S3.** PTM levels in cheeses classified by animal species used for milk production

| **Milk source** | **Al^a^** | **Cr^b^** | **Fe^a^** | **Ni^b^** | **Cu^b^** | **Zn^a^** | **As^b^** | **Se^b^** | **Cd^b^** | **Pb^b^** |
| --- | --- | --- | --- | --- | --- | --- | --- | --- | --- | --- |
| **Cow milk** | | | | | | | | | | |
| <LOD% | - | - | - | - | - | - | - | - | - | - |
| <LOQ% | 11.1 | 85.2 | 51.9 | 85.2 | 81.5 | - | 100 | 92.6 | 100 | 96.3 |
| Min | <LOQ | <LOQ | <LOQ | <LOQ | <LOQ | 0.68 | <LOQ | <LOQ | <LOQ | <LOQ |
| Max | 7.29 | 25.4 | 5.95 | 64.9 | 4067 | 35.7 | <LOQ | 565 | <LOQ | 1754 |
| **Sheep milk** | | | | | | | | | | |
| <LOD% | - | - | - | - | - | - | - | - | - | - |
| <LOQ% | 26.7 | 73.3 | 13.3 | 46.7 | - | - | 100 | 93.3 | 93.3 | 100 |
| Min | <LOQ | <LOQ | <LOQ | <LOQ | 8.28 | 7.06 | <LOQ | <LOQ | <LOQ | <LOQ |
| Max | 4.76 | 244 | 5.89 | 108 | 506 | 27.0 | <LOQ | 730 | 8.69 | <LOQ |
| **Goat milk** | | | | | | | | | | |
| <LOD% | - | - | - | - | - | - | - | - | - | - |
| <LOQ% | 23.5 | 88.2 | 29.4 | 52.9 | 29.4 | - | 100 | 94.1 | 94.1 | 88.2 |
| Min | <LOQ | <LOQ | <LOQ | <LOQ | <LOQ | 2.38 | <LOQ | <LOQ | <LOQ | <LOQ |
| Max | 3.69 | 61.2 | 3.63 | 151 | 1394 | 56.6 | <LOQ | 1378 | 0.61 | 1281 |
| **Buffalo milk** | | | | | | | | | | |
| <LOD% | - | - | - | - | - | - | - | - | - | - |
| <LOQ% | 22.2 | 88.9 | 11.1 | 44.4 | 11.1 | - | 100 | 88.9 | 66.7 | 100 |
| Min | <LOQ | <LOQ | <LOQ | <LOQ | <LOQ | 9.07 | <LOQ | <LOQ | <LOQ | <LOQ |
| Max | 5.89 | 20.8 | 3.30 | 131 | 556 | 25.8 | <LOQ | 407 | 8.69 | <LOQ |
| **Mixed (Cow+Sheep+Goat+Buffalo)** | | | | | | | | | | |
| <LOD% | - | - | - | - | - | - | - | - | - | - |
| <LOQ% | - | 81.3 | 18.8 | 31.3 | 12.5 | - | 100 | 100 | 87.5 | 93.8 |
| Min | 0.21 | <LOQ | <LOQ | <LOQ | <LOQ | 10.7 | <LOQ | <LOQ | <LOQ | <LOQ |
| Max | 5.47 | 87.8 | 10.8 | 369 | 2075 | 32.2 | <LOQ | <LOQ | 12.3 | 93.5 |

^a^ mg/kg dry weight, ^b^ µg/kg dry weight, LOD: Limit of detection, LOQ: Limit of quantification.

**Table S4.** PTM levels in cheeses classified by production type

| **Production type** | **Al^a^** | **Cr^b^** | **Fe^a^** | **Ni^b^** | **Cu^b^** | **Zn^a^** | **As^b^** | **Se^b^** | **Cd^b^** | **Pb^b^** |
| --- | --- | --- | --- | --- | --- | --- | --- | --- | --- | --- |
| **Industrial** | | | | | | | | | | |
| <LOD% | - | - | - | - | - | - | - | - | - | - |
| <LOQ% | 17.9 | 71.8 | 43.6 | 61.5 | 12.8 | - | 100 | 94.9 | 92.3 | 89.7 |
| Min | <LOQ | <LOQ | <LOQ | <LOQ | <LOQ | 5.41 | <LOQ | <LOQ | <LOQ | <LOQ |
| Max | 7.29 | 244 | 38.8 | 369 | 2075 | 33.4 | <LOQ | 565 | 12.3 | 93.5 |
| **Artisanal** | | | | | | | | | | |
| <LOD% | - | - | - | - | - | - | - | - | - | - |
| <LOQ% | 11.1 | 93.3 | 37.8 | 71.1 | 20.0 | - | 100 | 97.8 | 93.3 | 93.3 |
| Min | <LOQ | <LOQ | <LOQ | <LOQ | <LOQ | 0.68 | <LOQ | <LOQ | <LOQ | <LOQ |
| Max | 4.76 | 61.2 | 5.89 | 151 | 4067 | 56.6 | <LOQ | 1378 | 0.61 | 1754 |

^a^ mg/kg dry weight, ^b^ µg/kg dry weight, LOD: Limit of detection, LOQ: Limit of quantification.

**Table S5.** PTM levels in cheeses classified by region

| **Regions** | **Al^a^** | **Cr^b^** | **Fe^a^** | **Ni^b^** | **Cu^b^** | **Zn^a^** | **As^b^** | **Se^b^** | **Cd^b^** | **Pb^b^** |
| --- | --- | --- | --- | --- | --- | --- | --- | --- | --- | --- |
| **EAR** | | | | | | | | | | |
| <LOD% | - | - | - | - | - | - | - | - | - | - |
| <LOQ% | - | 78.6 | 7.1 | 71.4 | 21.4 | 0.0 | 100 | 71.4 | 100 | 100 |
| Min | 0.07 | <LOQ | <LOQ | <LOQ | <LOQ | 7.04 | <LOQ | <LOQ | <LOQ | <LOQ |
| Max | 5.47 | 87.8 | 6.58 | 369 | 2075 | 33.4 | <LOQ | 1378 | <LOQ | <LOQ |
| **MR** | | | | | | | | | | |
| <LOD% | - | - | - | - | - | - | - | - | - | - |
| <LOQ% | 31.3 | 62.5 | 18.8 | 50.0 | 100 | - | 100 | 100 | 93.8 | 87.5 |
| Min | <LOQ | <LOQ | <LOQ | <LOQ | 8.28 | 13.4 | <LOQ | <LOQ | 0.00 | 0.00 |
| Max | 2.53 | 31.3 | 38.8 | 108 | 556 | 32.2 | <LOQ | <LOQ | 12.3 | 93.5 |
| **AR** | | | | | | | | | | |
| <LOD% | - | - | - | - | - | - | - | - | - | - |
| <LOQ% | 33.3 | 88.9 | 44.4 | 77.8 | 77.8 | - | 100 | 100 | 100 | 100 |
| Min | <LOQ | <LOQ | <LOQ | <LOQ | <LOQ | 0.68 | <LOQ | <LOQ | <LOQ | <LOQ |
| Max | 2.69 | 244 | 2.83 | 103 | 25.5 | 26.3 | <LOQ | <LOQ | <LOQ | <LOQ |
| **MER** | | | | | | | | | | |
| <LOD% | - | - | - | - | - | - | - | - | - | - |
| <LOQ% | 25.0 | 75.0 | 50.0 | 75.0 | 12.5 | - | 100 | 100 | 100 | 100 |
| Min | <LOQ | <LOQ | <LOQ | <LOQ | <LOQ | 8.58 | <LOQ | <LOQ | <LOQ | <LOQ |
| Max | 2.47 | 61.2 | 1.63 | 151 | 1394 | 28.6 | <LOQ | <LOQ | <LOQ | <LOQ |
| **BSR** | | | | | | | | | | |
| <LOD% | - | - | - | - | - | - | - | - | - | - |
| <LOQ% | - | 100 | 54.5 | 81.8 | - | - | 100 | 100 | 90.9 | 81.8 |
| Min | 0.02 | <LOQ | <LOQ | <LOQ | 28.9 | 5.41 | <LOQ | <LOQ | <LOQ | 0.00 |
| Max | 5.89 | <LOQ | 1.62 | 41.3 | 4067 | 35.7 | <LOQ | <LOQ | 0.90 | 1754 |
| **CAR** | | | | | | | | | | |
| <LOD% | - | - | - | - | - | - | - | - | - | - |
| <LOQ% | 21.4 | 85.7 | 28.6 | 64.3 | - | - | 100 | 100 | 78.6 | 92.9 |
| Min | <LOQ | <LOQ | <LOQ | <LOQ | <LOQ | 8.66 | <LOQ | <LOQ | <LOQ | <LOQ |
| Max | 7.29 | 25.4 | 5.95 | 22.2 | 570 | 56.6 | <LOQ | <LOQ | 0.61 | 8.49 |
| **SAR** | | | | | | | | | | |
| <LOD% | - | - | - | - | - | - | - | - | - | - |
| <LOQ% | - | 100 | 27.3 | 63.6 | 9.1 | - | 100 | 90.9 | 100 | 100 |
| Min | 0.06 | <LOQ | <LOQ | <LOQ | <LOQ | 7.03 | <LOQ | <LOQ | <LOQ | <LOQ |
| Max | 4.32 | <LOQ | 5.89 | 28.7 | 506 | 21.1 | <LOQ | 730 | <LOQ | <LOQ |

^a^ mg/kg dry weight, ^b^ µg/kg dry weight, EAR: East Anatolia Region, MR: Marmara Region, AR: Aegean Region, MER: Mediterranean Region, BSR: Black Sea Region, CAR: Central Anatolia Region, SAR: Southeast Anatolia Region, LOD: Limit of detection, LOQ: Limit of quantification.

**Table S6.** PTM exposure levels resulting from the consumption of cheeses classified by ripening stage (µg/kg/day)

| **Ripening type** | **Al** | **Cr** | **Fe** | **Ni** | **Cu** | **Zn** | **As** | **Se** | **Cd** | **Pb** |
| --- | --- | --- | --- | --- | --- | --- | --- | --- | --- | --- |
| **Ripened** | | | | | | | | | | |
| Median | 0.44 | 0.00 | 0.82 | 0.00 | 0.07 | 10.1 | 0.00 | 0.00 | 0.00 | 0.00 |
| Min | 0.00 | 0.00 | 0.00 | 0.00 | 0.00 | 0.39 | 0.00 | 0.00 | 0.00 | 0.00 |
| Max | 4.17 | 0.02 | 6.17 | 0.06 | 0.32 | 19.1 | 0.00 | 0.42 | 0.01 | 0.05 |
| **Fresh** | | | | | | | | | | |
| Median | 0.65 | 0.00 | 0.10 | 0.00 | 0.05 | 9.71 | 0.00 | 0.00 | 0.00 | 0.00 |
| Min | 0.00 | 0.00 | 0.00 | 0.00 | 0.00 | 3.09 | 0.00 | 0.00 | 0.00 | 0.00 |
| Max | 4.11 | 0.14 | 21.9 | 0.21 | 2.32 | 32.3 | 0.00 | 0.79 | 0.00 | 1.00 |

**Table S7.** PTM exposure levels resulting from the consumption of cheeses classified by animal species used for milk production (µg/kg/day)

| **Milk source** | **Al** | **Cr** | **Fe** | **Ni** | **Cu** | **Zn** | **As** | **Se** | **Cd** | **Pb** |
| --- | --- | --- | --- | --- | --- | --- | --- | --- | --- | --- |
| **Cow milk** | | | | | | | | | | |
| Median | 0.41 | 0.00 | 0.00 | 0.00 | 0.05 | 9.71 | 0.00 | 0.00 | 0.00 | 0.00 |
| Min | 0.00 | 0.00 | 0.00 | 0.00 | 0.00 | 0.39 | 0.00 | 0.00 | 0.00 | 0.00 |
| Max | 4.17 | 0.01 | 3.40 | 0.04 | 2.32 | 20.4 | 0.00 | 0.32 | 0.00 | 1.00 |
| **Sheep milk** | | | | | | | | | | |
| Median | 0.51 | 0.00 | 0.93 | 0.00 | 0.09 | 9.31 | 0.00 | 0.00 | 0.00 | 0.00 |
| Min | 0.00 | 0.00 | 0.00 | 0.00 | 0.00 | 4.03 | 0.00 | 0.00 | 0.00 | 0.00 |
| Max | 2.72 | 0.14 | 3.37 | 0.06 | 0.29 | 15.4 | 0.00 | 0.42 | 0.00 | 0.00 |
| **Goat milk** | | | | | | | | | | |
| Median | 0.23 | 0.00 | 0.51 | 0.00 | 0.06 | 8.34 | 0.00 | 0.00 | 0.00 | 0.00 |
| Min | 0.00 | 0.00 | 0.00 | 0.00 | 0.00 | 1.36 | 0.00 | 0.00 | 0.00 | 0.00 |
| Max | 2.11 | 0.03 | 2.07 | 0.09 | 0.80 | 32.3 | 0.00 | 0.79 | 0.00 | 0.73 |
| **Buffalo milk** | | | | | | | | | | |
| Median | 0.96 | 0.00 | 0.23 | 0.00 | 0.04 | 9.77 | 0.00 | 0.00 | 0.00 | 0.00 |
| Min | 0.00 | 0.00 | 0.00 | 0.00 | 0.00 | 5.18 | 0.00 | 0.00 | 0.00 | 0.00 |
| Max | 3.37 | 0.01 | 1.89 | 0.07 | 0.32 | 14.7 | 0.00 | 0.23 | 0.00 | 0.00 |
| **Mixed (Cow+Sheep+Goat+Buffalo)** | | | | | | | | | | |
| Median | 0.87 | 0.00 | 1.17 | 0.00 | 0.06 | 11.7 | 0.00 | 0.00 | 0.00 | 0.00 |
| Min | 0.12 | 0.00 | 0.00 | 0.00 | 0.00 | 6.11 | 0.00 | 0.00 | 0.00 | 0.00 |
| Max | 3.13 | 0.05 | 6.17 | 0.21 | 1.19 | 18.4 | 0.00 | <0.00 | 0.01 | 0.05 |

**Table S8.** PTM exposure levels resulting from the consumption of cheeses classified by production type (µg/kg/day)

| **Production type** | **Al** | **Cr** | **Fe** | **Ni** | **Cu** | **Zn** | **As** | **Se** | **Cd** | **Pb** |  |
| --- | --- | --- | --- | --- | --- | --- | --- | --- | --- | --- | --- |
| **Industrial** | | | | | | | | | | | |
| Median | 0.87 | 0.00 | 0.82 | 0.00 | 0.07 | 11.0 | 0.00 | 0.00 | 0.00 | 0.00 |  |
| Min | 0.00 | 0.00 | 0.00 | 0.00 | 0.00 | 3.09 | 0.00 | 0.00 | 0.00 | 0.00 |  |
| Max | 4.17 | 0.14 | 22.2 | 0.21 | 1.19 | 19.1 | 0.00 | 0.32 | 0.01 | 0.05 |  |
| **Artisanal** | | | | | | | | | | | |
| Median | 0.42 | 0.00 | 0.19 | 0.00 | 0.05 | 9.14 | 0.00 | 0.00 | 0.00 | 0.00 |  |
| Min | 0.00 | 0.00 | 0.00 | 0.00 | 0.00 | 0.39 | 0.00 | 0.00 | 0.00 | 0.00 |  |
| Max | 2.72 | 0.03 | 3.37 | 0.09 | 2.32 | 32.3 | 0.00 | 0.79 | 0.00 | 1.00 |  |

**Table S9.** PTM exposure levels resulting from the consumption of cheeses classified by region (µg/kg/day)

| **Regions** | **Al** | **Cr** | **Fe** | **Ni** | **Cu** | **Zn** | **As** | **Se** | **Cd** | **Pb** |  |
| --- | --- | --- | --- | --- | --- | --- | --- | --- | --- | --- | --- |
| **EAR** | | | | | | | | | | | |
| Median | 0.75 | 0.00 | 0.77 | 0.00 | 0.05 | 9.77 | 0.00 | 0.00 | 0.00 | 0.00 |  |
| Min | 0.04 | 0.00 | 0.00 | 0.00 | 0.00 | 4.02 | 0.00 | 0.00 | 0.00 | 0.00 |  |
| Max | 3.13 | 0.05 | 3.76 | 0.21 | 1.19 | 19.1 | 0.00 | 0.79 | 0.00 | 0.00 |  |
| **MR** | | | | | | | | | | | |
| Median | 0.23 | 0.00 | 0.94 | 0.00 | 0.08 | 11.4 | 0.00 | 0.00 | 0.00 | 0.00 |  |
| Min | 0.00 | 0.00 | 0.00 | 0.00 | 0.00 | 7.66 | 0.00 | 0.00 | 0.00 | 0.00 |  |
| Max | 1.45 | 0.02 | 22.2 | 0.06 | 0.32 | 18.4 | 0.00 | <0.00 | 0.01 | 0.05 |  |
| **AR** | | | | | | | | | | | |
| Median | 0.35 | 0.00 | 0.08 | 0.00 | 0.00 | 5.71 | 0.00 | 0.00 | 0.00 | 0.00 |  |
| Min | 0.00 | 0.00 | 0.00 | 0.00 | 0.00 | 0.39 | 0.00 | 0.00 | 0.00 | 0.00 |  |
| Max | 1.54 | 0.14 | 1.62 | 0.06 | 0.01 | 15.0 | 0.00 | 0.00 | 0.00 | 0.00 |  |
| **MER** | | | | | | | | | | | |
| Median | 0.28 | 0.00 | 0.02 | 0.00 | 0.06 | 12.2 | 0.00 | 0.00 | 0.00 | 0.00 |  |
| Min | 0.00 | 0.00 | 0.00 | 0.00 | 0.00 | 4.90 | 0.00 | 0.00 | 0.00 | 0.00 |  |
| Max | 1.41 | 0.03 | 0.93 | 0.09 | 0.80 | 16.3 | 0.00 | 0.00 | 0.00 | 0.00 |  |
| **BSR** | | | | | | | | | | | |
| Median | 0.90 | 0.00 | 0.00 | 0.00 | 0.10 | 12.9 | 0.00 | 0.00 | 0.00 | 0.00 |  |
| Min | 0.01 | 0.00 | 0.00 | 0.00 | 0.02 | 3.09 | 0.00 | 0.00 | 0.00 | 0.00 |  |
| Max | 3.37 | 0.00 | 0.93 | 0.02 | 2.32 | 20.4 | 0.00 | 0.00 | <0.00 | 1.00 |  |
| **CAR** | | | | | | | | | | | |
| Median | 0.37 | 0.00 | 0.85 | 0.00 | 0.06 | 7.94 | 0.00 | 0.00 | 0.00 | 0.00 |  |
| Min | 0.00 | 0.00 | 0.00 | 0.00 | 0.00 | 4.95 | 0.00 | 0.00 | 0.00 | 0.00 |  |
| Max | 4.17 | 0.01 | 3.40 | 0.01 | 0.33 | 32.3 | 0.00 | 0.00 | 0.00 | 0.00 |  |
| **SAR** | | | | | | | | | | | |
| Median | 0.95 | 0.00 | 1.03 | 0.00 | 0.08 | 9.49 | 0.00 | 0.00 | 0.00 | 0.00 |  |
| Min | 0.03 | 0.00 | 0.00 | 0.00 | 0.00 | 4.02 | 0.00 | 0.00 | 0.00 | 0.00 |  |
| Max | 2.47 | 0.00 | 3.37 | 0.02 | 0.29 | 12.1 | 0.00 | 0.42 | 0.00 | 0.00 |  |

EAR: East Anatolia Region, MR: Marmara Region, AR: Aegean Region, MER: Mediterranean Region, BSR: Black Sea Region, CAR: Central Anatolia Region, SAR: Southeast Anatolia Region.

**Table S10.** Toxicological contribution (%) rate of PTM exposure level from cheese consumption

|  |  | **Al** | **Cr (F)** | **Cr (M)** | **Fe (F)** | **Fe (M)** | **Ni** | **Cu** | **Zn (F)** | **Zn (M)** | **Se** | **Cd** |
| --- | --- | --- | --- | --- | --- | --- | --- | --- | --- | --- | --- | --- |
| **All cheese** | Mean | 0.27 | 0.02 | 0.01 | 0.42 | 0.94 | 0.07 | 1.00 | 6.55 | 9.02 | 2.06 | 0.02 |
|  | Max | 1.46 | 0.56 | 0.40 | 8.63 | 19.4 | 1.62 | 17.9 | 20.6 | 28.4 | 99.7 | 0.70 |
| **Ripening type** | | | | | | | | | | | | |
| Ripened | Mean | 0.26 | 0.01 | 0.01 | 0.40 | 0.90 | 0.04 | 0.67 | 6.72 | 9.25 | 2.15 | 0.03 |
|  | Max | 1.46 | 0.07 | 0.05 | 2.40 | 5.41 | 0.48 | 2.45 | 12.2 | 16.7 | 52.8 | 0.70 |
| Fresh | Mean | 0.30 | 0.02 | 0.02 | 0.42 | 0.95 | 0.12 | 1.44 | 6.47 | 8.91 | 3.31 | 0.00 |
|  | Max | 1.44 | 0.56 | 0.40 | 8.52 | 19.2 | 1.62 | 17.9 | 20.6 | 28.4 | 99.7 | 0.05 |
| **Milk source** | | | | | | | | | | | | |
| Cow milk | Mean | 0.31 | 0.00 | 0.00 | 0.20 | 0.44 | 0.02 | 1.28 | 6.70 | 9.22 | 1.63 | 0.00 |
|  | Max | 1.46 | 0.06 | 0.04 | 1.32 | 2.98 | 0.29 | 17.9 | 13.0 | 17.9 | 40.9 | 0.00 |
| Sheep milk | Mean | 0.25 | 0.05 | 0.04 | 0.39 | 0.89 | 0.10 | 0.83 | 6.23 | 8.59 | 3.52 | 0.03 |
|  | Max | 0.95 | 0.56 | 0.40 | 1.31 | 2.95 | 0.48 | 2.22 | 9.83 | 13.5 | 52.8 | 0.50 |
| Goat milk | Mean | 0.18 | 0.01 | 0.01 | 0.22 | 0.50 | 0.06 | 0.92 | 6.01 | 8.27 | 5.86 | 0.00 |
|  | Max | 0.74 | 0.14 | 0.10 | 0.81 | 1.82 | 0.66 | 6.13 | 20.6 | 28.4 | 99.7 | 0.04 |
| Buffalo milk | Mean | 0.36 | 0.01 | 0.00 | 0.20 | 0.44 | 0.13 | 0.57 | 6.35 | 8.74 | 3.27 | 0.06 |
|  | Max | 1.18 | 0.05 | 0.03 | 0.73 | 1.65 | 0.58 | 2.45 | 9.39 | 12.9 | 29.4 | 0.50 |
| Mixed  (Cow+Sheep+Goat+Buffalo) | Mean | 0.34 | 0.02 | 0.01 | 0.58 | 1.30 | 0.11 | 1.14 | 7.51 | 10.3 | 0.00 | 0.05 |
|  | Max | 1.09 | 0.20 | 0.14 | 2.40 | 5.41 | 1.62 | 9.12 | 11.7 | 16.1 | 0.00 | 0.70 |
| **Production type** | | | | | | | | | | | | |
| Industrial | Mean | 0.33 | 0.03 | 0.02 | 0.65 | 1.47 | 0.11 | 0.78 | 6.99 | 9.63 | 1.89 | 0.03 |
|  | Max | 1.46 | 0.56 | 0.40 | 8.63 | 19.4 | 1.62 | 9.12 | 12.2 | 16.7 | 40.9 | 0.70 |
| Artisanal | Mean | 0.21 | 0.00 | 0.00 | 0.21 | 0.48 | 0.04 | 1.19 | 6.17 | 8.49 | 2.21 | 0.00 |
|  | Max | 0.95 | 0.14 | 0.10 | 1.31 | 2.95 | 0.66 | 17.9 | 20.6 | 28.4 | 99.7 | 0.04 |
| **Region** | | | | | | | | | | | | |
| EAR | Mean | 0.39 | 0.02 | 0.02 | 0.44 | 0.99 | 0.17 | 1.12 | 6.91 | 9.51 | 12.4 | 0.00 |
|  | Max | 1.09 | 0.20 | 0.14 | 1.46 | 3.30 | 1.62 | 9.12 | 12.2 | 16.7 | 99.7 | 0.00 |
| MR | Mean | 0.14 | 0.01 | 0.01 | 0.95 | 2.13 | 0.11 | 0.81 | 7.36 | 10.1 | 0.00 | 0.07 |
|  | Max | 0.51 | 0.07 | 0.05 | 8.63 | 19.4 | 0.48 | 2.45 | 11.7 | 16.1 | 0.00 | 0.70 |
| AR | Mean | 0.17 | 0.06 | 0.04 | 0.12 | 0.26 | 0.05 | 0.02 | 3.97 | 5.47 | 0.00 | 0.00 |
|  | Max | 0.54 | 0.56 | 0.40 | 0.63 | 1.42 | 0.45 | 0.11 | 9.57 | 13.2 | 0.00 | 0.00 |
| MER | Mean | 0.15 | 0.02 | 0.01 | 0.07 | 0.17 | 0.11 | 1.23 | 7.25 | 9.98 | 0.00 | 0.00 |
|  | Max | 0.49 | 0.14 | 0.10 | 0.36 | 0.82 | 0.66 | 6.13 | 10.4 | 14.3 | 0.00 | 0.00 |
| BSR | Mean | 0.35 | 0.00 | 0.00 | 0.08 | 0.17 | 0.02 | 2.66 | 7.47 | 10.3 | 0.00 | 0.00 |
|  | Max | 1.18 | 0.00 | 0.00 | 0.36 | 0.81 | 0.18 | 17.9 | 13.0 | 17.9 | 0.00 | 0.05 |
| CAR | Mean | 0.29 | 0.01 | 0.01 | 0.37 | 0.83 | 0.02 | 0.71 | 6.71 | 9.24 | 0.00 | 0.01 |
|  | Max | 1.46 | 0.06 | 0.04 | 1.32 | 2.98 | 0.10 | 2.51 | 20.6 | 28.4 | 0.00 | 0.04 |
| SAR | Mean | 0.37 | 0.00 | 0.00 | 0.43 | 0.97 | 0.02 | 0.67 | 5.72 | 7.88 | 4.80 | 0.00 |
|  | Max | 0.86 | 0.00 | 0.00 | 1.31 | 2.95 | 0.13 | 2.22 | 7.68 | 10.6 | 52.8 | 0.00 |

EAR: East Anatolia Region, MR: Marmara Region, AR: Aegean Region, MER: Mediterranean Region, BSR: Black Sea Region, CAR: Central Anatolia Region, SAR: Southeast Anatolia Region. F: Female, M: Male.

**Table S11.** THQ values of cheeses classified by ripening stage

| **Ripening type** | **Al** | **Cr** | **Fe** | **Ni** | **Cu** | **Zn** | **As** | **Se** | **Cd** | **Pb** |
| --- | --- | --- | --- | --- | --- | --- | --- | --- | --- | --- |
| **Ripened** | | | | | | | | | | |
| Median | <0.00 | 0.00 | <0.00 | 0.00 | <0.00 | 0.03 | 0.00 | 0.00 | 0.00 | 0.00 |
| Min | 0.00 | 0.00 | 0.00 | 0.00 | 0.00 | <0.00 | 0.00 | 0.00 | 0.00 | 0.00 |
| Max | 0.03 | 0.01 | 0.01 | <0.00 | 0.01 | 0.06 | 0.00 | 0.08 | 0.01 | 0.01 |
| **Fresh** | | | | | | | | | | |
| Median | <0.00 | 0.00 | <0.00 | 0.00 | <0.00 | 0.03 | 0.00 | 0.00 | 0.00 | 0.00 |
| Min | 0.00 | 0.00 | 0.00 | 0.00 | 0.00 | 0.01 | 0.00 | 0.00 | 0.00 | 0.00 |
| Max | 0.03 | 0.05 | 0.03 | 0.01 | 0.06 | 0.11 | 0.00 | 0.16 | 0.00 | 0.25 |

**Table S12**. THQ values of cheeses classified by animal species used for milk production

| **Milk source** | **Al** | **Cr** | **Fe** | **Ni** | **Cu** | **Zn** | **As** | **Se** | **Cd** | **Pb** |
| --- | --- | --- | --- | --- | --- | --- | --- | --- | --- | --- |
| **Cow milk** | | | | | | | | | | |
| Median | <0.00 | 0.00 | 0.00 | 0.00 | <0.00 | 0.03 | 0.00 | 0.00 | 0.00 | 0.00 |
| Min | 0.00 | 0.00 | 0.00 | 0.00 | 0.00 | <0.00 | 0.00 | 0.00 | 0.00 | 0.00 |
| Max | 0.03 | <0.00 | <0.00 | <0.00 | 0.06 | 0.07 | 0.00 | 0.06 | 0.00 | 0.25 |
| **Sheep milk** | | | | | | | | | | |
| Median | <0.00 | 0.00 | <0.00 | 0.00 | <0.00 | 0.03 | 0.00 | 0.00 | 0.00 | 0.00 |
| Min | 0.00 | 0.00 | 0.00 | 0.00 | 0.00 | 0.01 | 0.00 | 0.00 | 0.00 | 0.00 |
| Max | 0.02 | 0.05 | <0.00 | <0.00 | 0.01 | 0.05 | 0.00 | 0.08 | 0.00 | 0.00 |
| **Goat milk** | | | | | | | | | | |
| Median | <0.00 | 0.00 | <0.00 | 0.00 | <0.00 | 0.03 | 0.00 | 0.00 | 0.00 | 0.00 |
| Min | 0.00 | 0.00 | 0.00 | 0.00 | 0.00 | <0.00 | 0.00 | 0.00 | 0.00 | 0.00 |
| Max | 0.01 | 0.01 | <0.00 | <0.00 | 0.02 | 0.11 | 0.00 | 0.16 | 0.00 | 0.18 |
| **Buffalo milk** | | | | | | | | | | |
| Median | 0.01 | 0.00 | <0.00 | 0.00 | <0.00 | 0.03 | 0.00 | 0.00 | 0.00 | 0.00 |
| Min | 0.00 | 0.00 | 0.00 | 0.00 | 0.00 | 0.02 | 0.00 | 0.00 | 0.00 | 0.00 |
| Max | 0.02 | <0.00 | <0.00 | <0.00 | <0.01 | 0.05 | 0.00 | 0.05 | 0.00 | 0.00 |
| **Mixed (cow+sheep+goat+buffalo)** | | | | | | | | | | |
| Median | 0.01 | 0.00 | <0.00 | 0.00 | <0.00 | 0.04 | 0.00 | 0.00 | 0.00 | 0.00 |
| Min | <0.00 | 0.00 | 0.00 | 0.00 | 0.00 | 0.02 | 0.00 | 0.00 | 0.00 | 0.00 |
| Max | 0.02 | 0.02 | 0.01 | 0.01 | 0.03 | 0.06 | 0.00 | 0.00 | 0.01 | 0.01 |

**Table S13.** THQ values of cheeses classified by production type

| **Production type** | **Al** | **Cr** | **Fe** | **Ni** | **Cu** | **Zn** | **As** | **Se** | **Cd** | **Pb** |
| --- | --- | --- | --- | --- | --- | --- | --- | --- | --- | --- |
| **Industrial** | | | | | | | | | | |
| Median | 0.01 | 0.00 | <0.00 | 0.00 | <0.00 | 0.04 | 0.00 | 0.00 | 0.00 | 0.00 |
| Min | 0.00 | 0.00 | 0.00 | 0.00 | 0.00 | 0.01 | 0.00 | 0.00 | 0.00 | 0.00 |
| Max | 0.03 | 0.05 | 0.03 | 0.01 | 0.03 | 0.06 | 0.00 | 0.06 | 0.01 | 0.01 |
| **Artisanal** | | | | | | | | | | |
| Median | <0.00 | 0.00 | <0.00 | 0.00 | <0.00 | 0.03 | 0.00 | 0.00 | 0.00 | 0.00 |
| Min | 0.00 | 0.00 | 0.00 | 0.00 | 0.00 | <0.00 | 0.00 | 0.00 | 0.00 | 0.00 |
| Max | 0.02 | 0.01 | <0.00 | <0.00 | 0.06 | 0.11 | 0.00 | 0.16 | 0.00 | 0.25 |

**Table S14.** THQ values of cheeses classified by region

| **Regions** | **Al** | **Cr** | **Fe** | **Ni** | **Cu** | **Zn** | **As** | **Se** | **Cd** | **Pb** |
| --- | --- | --- | --- | --- | --- | --- | --- | --- | --- | --- |
| **EAR** | | | | | | | | | | |
| Median | 0.01 | 0.00 | <0.00 | 0.00 | <0.00 | 0.03 | 0.00 | 0.00 | 0.00 | 0.00 |
| Min | 0.00 | 0.00 | 0.00 | 0.00 | 0.00 | 0.01 | 0.00 | 0.00 | 0.00 | 0.00 |
| Max | 0.02 | 0.02 | 0.01 | 0.01 | 0.03 | 0.06 | 0.00 | 0.16 | 0.00 | 0.00 |
| **MR** | | | | | | | | | | |
| Median | <0.00 | 0.00 | <0.00 | 0.00 | <0.00 | 0.04 | 0.00 | 0.00 | 0.00 | 0.00 |
| Min | 0.00 | 0.00 | 0.00 | 0.00 | 0.00 | 0.03 | 0.00 | 0.00 | 0.00 | 0.00 |
| Max | 0.01 | 0.01 | 0.03 | <0.00 | 0.01 | 0.06 | 0.00 | 0.00 | 0.01 | 0.01 |
| **AR** | | | | | | | | | | |
| Median | <0.00 | 0.00 | <0.00 | 0.00 | 0.00 | 0.02 | 0.00 | 0.00 | 0.00 | 0.00 |
| Min | 0.00 | 0.00 | 0.00 | 0.00 | 0.00 | 0.00 | 0.00 | 0.00 | 0.00 | 0.00 |
| Max | 0.01 | 0.05 | <0.00 | <0.00 | <0.00 | 0.05 | 0.00 | 0.00 | 0.00 | 0.00 |
| **MER** | | | | | | | | | | |
| Median | <0.00 | 0.00 | <0.00 | 0.00 | <0.00 | 0.04 | 0.00 | 0.00 | 0.00 | 0.00 |
| Min | 0.00 | 0.00 | 0.00 | 0.00 | 0.00 | 0.02 | 0.00 | 0.00 | 0.00 | 0.00 |
| Max | 0.01 | 0.01 | <0.00 | <0.00 | 0.02 | 0.05 | 0.00 | 0.00 | 0.00 | 0.00 |
| **BSR** | | | | | | | | | | |
| Median | 0.01 | 0.00 | 0.00 | 0.00 | <0.00 | 0.04 | 0.00 | 0.00 | 0.00 | 0.00 |
| Min | <0.00 | 0.00 | 0.00 | 0.00 | <0.00 | 0.01 | 0.00 | 0.00 | 0.00 | 0.00 |
| Max | 0.02 | 0.00 | <0.00 | <0.00 | 0.06 | 0.07 | 0.00 | 0.00 | 0.00 | 0.25 |
| **CAR** | | | | | | | | | | |
| Median | <0.00 | 0.00 | <0.00 | 0.00 | <0.00 | 0.03 | 0.00 | 0.00 | 0.00 | 0.00 |
| Min | 0.00 | 0.00 | 0.00 | 0.00 | 0.00 | 0.02 | 0.00 | 0.00 | 0.00 | 0.00 |
| Max | 0.03 | <0.00 | <0.00 | <0.00 | 0.01 | 0.11 | 0.00 | 0.00 | 0.00 | 0.00 |
| **SAR** | | | | | | | | | | |
| Median | 0.01 | 0.00 | <0.00 | 0.00 | <0.00 | 0.03 | 0.00 | 0.00 | 0.00 | 0.00 |
| Min | <0.00 | 0.00 | 0.00 | 0.00 | 0.00 | 0.01 | 0.00 | 0.00 | 0.00 | 0.00 |
| Max | 0.02 | 0.00 | <0.00 | <0.00 | 0.01 | 0.04 | 0.00 | 0.08 | 0.00 | 0.00 |

EAR: East Anatolia Region, MR: Marmara Region, AR: Aegean Region, MER: Mediterranean Region, BSR: Black Sea Region, CAR: Central Anatolia Region, SAR: Southeast Anatolia Region.

**Figure S1.** PCA score plot of cheese samples according to ripening stage

**Figure S2.** PCA score plot of cheese samples according to milk source

**Figure S3.** PCA score plot of cheese samples according to production type

**Figure S4.** PCA score plot of cheese samples according to geographical region

**Figure S5.** PCA loading plot for Al, Fe, Ni, Cu, and Zn

**Figure S6.** Scree plot of the PCA model
